# Participation in After-School Extracurricular Activities and Cognitive Ability Among Early Adolescents in China: Moderating Effects of Gender and Family Economic Status

**DOI:** 10.3389/fped.2022.839473

**Published:** 2022-03-17

**Authors:** Yangu Pan, Di Zhou, Daniel Tan Lei Shek

**Affiliations:** ^1^Research Institute of Social Development, Southwestern University of Finance and Economics, Chengdu, China; ^2^Department of Applied Social Sciences, Hong Kong Polytechnic University, Kowloon, Hong Kong SAR, China

**Keywords:** extracurricular activities, cognitive ability, gender, family economic status, early adolescents, China

## Abstract

Although theories and research suggest that participation in extracurricular activities plays an important role in adolescents’ cognitive development, few studies have addressed this issue among early adolescents in China. Based on the responses of 9,830 Chinese junior high school students (*M*_age_ = 14.54 years, *SD* = 0.70 years), we investigated the relationships between different types of extracurricular activities and cognitive ability among junior high school students and the moderating effects of gender and family economic status. Using multi-level multiple regression analyses, results indicated that while time spent completing homework and physical exercise was positively associated with students’ cognitive ability, time spent on extracurricular tutoring, interest classes, watching TV, and surfing online and playing games was negatively related to students’ cognitive ability. The observed relationships were also moderated by gender and family economic status. Specifically, time spent on completing homework had a stronger positive relationship with boys’ cognitive ability, whereas time spent attending extracurricular tutoring on weekdays had a stronger negative relationship with girls’ cognitive ability, and time spent on physical exercise was more strongly related to girls’ cognitive ability in a positive manner. Besides, time spent attending interest classes on weekdays had a stronger negative relationship with cognitive ability among students from wealthy families, and time spent watching TV and physical exercise had stronger negative and positive effects on the cognitive ability among students from economically disadvantaged families, respectively. The theoretical and practical implications of the findings regarding the role of extra-curricular activities on adolescent development are discussed.

## Introduction

The development of cognitive ability is an important part of adolescent development. Cognitive ability has been found to be positively associated with higher educational attainment and wages (1–3), better workplace performance (1), and reduced risk of mental health problems (4–6). As such, it is important to identify risk and protective factors of adolescents’ cognitive development. According to Bronfenbrenner (7) social ecology theory, as a micro-ecosystem, after-school extracurricular activities plays an important role in adolescent development. Moreover, social capital theory suggests that extra-curricular activities have positive impacts on adolescent educational outcomes by providing sources of social control, as well as emotional and personal support (8). Empirical studies also indicated that extra-curricular activities have positive impacts on student’s behavior, school performance, and school completion (9, 10).

As Chinese adolescents constitute a significant proportion of world’s youth population, there is a need to understand the role of after-school extra-curricular activities on Chinese adolescent development. Since the 1990s, the Chinese government has been committed to reducing the learning burden of primary and secondary school students, hoping to provide them with a more relaxed and free extracurricular life. However, the latest survey report on Chinese children and adolescents (11) showed that compared with those born in the 90s, the post-00s generation had a heavier learning burden, and the time spent at school, homework, and extracurricular classes had increased significantly. Moreover, in the limited leisure time of adolescents, sedentary activities such as reading extracurricular books, watching TV, surfing the Internet and listening to songs were most popular, and the amount of physical exercise was generally insufficient (11). Recently, the Ministry of Education of China has issued a policy of “double reduction” to reduce homework burden and off-campus training burden on students during compulsory education, which would increase the free time available to students. Although students’ participation in different after-school extracurricular activities in free time may have different effects on their cognitive development, few studies have systematically investigated this issue among early adolescents in China. Therefore, it is necessary to explore the relationships between different extracurricular activities and adolescents’ cognitive ability and the related moderating factors.

### Extracurricular Activities and Cognitive Ability

There are many possible after-school extracurricular activities, ranging from highly structured activities (e.g., club activities) to relatively unstructured activities (e.g., surfing online) (12). The after-school extracurricular activities among junior high school students in China mainly include completing homework, attending extracurricular tutoring, interest classes, watching TV, surfing the Internet, and physical exercise (13, 14).

Regarding the relationship between after-school extracurricular learning activities and cognitive ability, the role of homework in educational outcomes has a long lasting debate (15). Generally speaking, homework is an activity that students do at home after school. In many studies based on Chinese students, “doing homework” was considered as students’ extracurricular activity (11, 16, 17). Teachers and parents tended to believe that homework time is positively associated with academic performance (18–21). However, some studies found that time spent on homework had little effect on academic performance (22, 23), and some even found a negative relationship (24, 25). Moreover, extracurricular tutoring (i.e., shadow education) is very common (26). Similarly, regarding the effectiveness of off-campus tutoring, previous studies found inconsistent and even contradictory results (26). While some found that participation in extracurricular tutoring was positively related to students’ development (27–29), other studies showed that there were few positive effects from participating in extracurricular tutoring (30). According to Bray (26), differences of these findings may be related to the different formats, delivery mechanisms and intensity of homework and extracurricular tutoring.

In addition to extracurricular tutoring, participating in interest-oriented classes is also very popular among Chinese children and adolescents, which generally refers to art education such as music, dance, painting, performance and so on. Although few studies have directly investigated the relationship between participation in interest classes and cognitive ability, some studies showed the positive influences of long-term art education such as painting, music and dance on cognitive abilities (31, 32). For example, Liu et al. (33) argued that art education could cultivate students’ physical functions, observation, imagination, creativity, and “healthy” personality.

In term of extracurricular entertainment activities, negative consequences of excessive watching TV and Internet use in adolescents were reported. For instance, Johnson et al. (34) found that three or more hours on watching television daily in adolescence was linked to attention problems, negative attitudes toward school, and poor academic performance. A review study suggests that Internet addiction could change adolescents’ brain structure (35–37) and might impair their cognitive function (38).

On the other hand, physical exercise was shown to have a positive effect on individual cognitive ability in previous studies (39–42). These include enhancing the nerve growth in brain regions related to cognition and memory (43), improving adolescents’ executive function and attention (44), and improving adolescents’ physical and mental health (45).

### Moderating Effect of Gender on the Association Between Extracurricular Activities and Cognitive Ability

Adolescent gender could moderate the association between extracurricular learning activities and cognitive ability, which may be due to gender differences in academic effort (46). Many researchers suggest that girls show more academic effort than do boys. For example, Lam et al. (47) investigated the topic based on a large number of adolescents and found that girls scored higher on persistence and effort. Male middle school students also showed higher effort avoidance than did female students in South Korea (48). Besides teachers, parents also considered girls as more self-disciplined than were boys (49). Thus, girls may get more cognitive improvement than boys when participating in the same learning activities.

In addition, interests of male and female students are different. Studies have found that boys are more likely to engage in playing video games or computer activities relative to girls (50–52). Moreover, previous studies indicated that excessive watching television impaired cognitive ability and reduced high school graduation rates for boys, but there was no such effect for girls (53). These results suggest that relative to female junior high school students, male students would be more likely to be negatively influenced by the Internet and television. Additionally, with regard to gender differences in the influence of physical exercise on students’ cognitive ability, Fang (40) found that although male and female students get similar benefit on cognitive ability from physical exercise, male students get higher benefit on academic achievements than did female students.

### Moderating Effect of Family Economic Status on the Association Between Extracurricular Activities and Cognitive Ability

Family economic status of the students is another factor that could impact the association between after-school extracurricular activities and cognitive ability. The “theory of faucet” proposed by Entwisle et al. (54) highlights the importance of family background to students’ participation in extracurricular activities. School is usually closed during the summer vacation, which is as the same as closing the “faucet” of school education, so that the differences of academic achievement of students might also depend on family background and social environment (55). Students from families with higher socioeconomic status are more likely to rely on family advantage to fill the education of extracurricular life, such as attending extracurricular tutoring (56), while students with lower socioeconomic status tend to spend more time on games and other entertainment activities in their extracurricular life which do not cost much (57). Moreover, Buchmann et al. (29) found that students from wealthier families could afford expensive off-campus tutoring to gain an advantage in university admission. Thus, relative to adolescents with lower family economic status, those with higher family economic status could obtain more improvement through participation in high-quality extracurricular tutoring. However, some studies suggested that students with lower family economic status may benefit more from extracurricular learning activities, because it provides some new opportunities to train their cognitive and non-cognitive abilities, which may be “over-excessive” for students with higher socioeconomic status (8, 58). For physical exercise, Fang (40) found that physical exercise improved the cognitive ability and academic achievements of students in low and middle-income families but not in high-income families.

### The Present Study

Although previous studies demonstrated that extra-curricular activities have some relationships with adolescent development, there are several limitations of the literature. First, previous studies on adolescents’ participation in extracurricular activities generally focused on structured extracurricular activities in school, with few studies exploring the effects of after-school unstructured extracurricular activities on adolescent development. Second, although previous studies mainly investigated the positive impacts of extracurricular activities on adolescents’ academic performance or social emotional development, few studies investigated the positive impacts of extracurricular activities on adolescents’ cognitive ability. Third, few studies investigated the role of extracurricular activities in adolescents’ development in non-Western culture such as China. Finally, the moderating effects of adolescents’ gender and family economic status on the association between extracurricular activities and adolescents’ development has rarely been examined. Therefore, the current study examined the relationship between participation in after-school extracurricular activities and students’ cognitive ability among Chinese adolescents, and the moderating effects of gender and family economic status. Specifically, we asked the following research questions in this study.

1. Is participation in different after-school extracurricular activities related to cognitive ability among early adolescents in China? Based on the literature reviewed above, we proposed that time spent on homework, extracurricular tutoring, interest classes and physical exercise would be positively associated with cognitive ability among junior high school students (Hypothesis 1a) and time spent on watching TV, surfing the Internet and playing games would be negatively associated with cognitive ability among junior high school students (Hypothesis 1b).
2. Does gender moderate the relationship between extracurricular activities and cognitive ability among Chinese early adolescents? With reference to the literature (40, 46, 50, 53), we proposed that time spent on homework, extracurricular tutoring and interest classes would be more strongly related to the cognitive ability among girls than boys in a positive manner (Hypothesis 2a). Besides, time spent on watching TV, surfing online and playing online games would be more strongly associated with cognitive ability in boys than girls in a negative manner (Hypothesis 2b). Finally, the positive relationship between the duration of extracurricular physical exercise and cognitive ability would be stronger in boys than in girls (Hypothesis 2c).
3. Does adolescents’ family economic status moderate the relationship between extracurricular activities and cognitive ability among early adolescents in China? Based on the literature, we proposed three hypotheses. First, relative to adolescents with high family economic status, time spent on homework, extracurricular tutoring and interest classes would have stronger positive relationship with cognitive ability among students with low family economic status (Hypothesis 3a). Second, time spent on watching TV and playing games online would have a stronger negative relationship with cognitive ability among students with low family economic status than did students with high family economic status (Hypothesis 3b). Third, time spent on physical exercise would have a stronger positive relationship with cognitive ability among students with low family economic status than did students with high family economic status (Hypothesis 3c).

## Materials and Methods

### Participants and Procedure

The data used in this study were drawn from the China Education Panel Survey (CEPS) designed by Renmin University of China which is a nationally representative survey of junior high school students. In this project, a stratified multistage sampling method was adopted, and participants were randomly selected from 438 classes of 112 junior high schools in 28 county-level districts in mainland China. A total of 9,920 students in classes (Grade 8) participated in 2014–2015. Participants completed a self-report questionnaire in class at school under the guidance of trained research assistants.

After we removed cases who had missing data for cognitive ability, there were 9,830 students (*M*_age_ = 14.54 years, *SD* = 0.70 years) in the final sample. Among them, there were 4,405 girls and 4,791 boys, and 634 students did not report their gender. Regarding “Hukou” (i.e., legal residence in a place), there were 4,910 students with rural Hukou, 4,456 students with urban Hukou, and 464 students’ Hukou information was missing. For family financial situation, 1,964 students were from families with reported “financial difficulty: (19.98%), 6,832 students were from families with reported “fine” economic status (69.50%), and 548 students were from reported “wealthy” families (5.57%), and 486 students did not report their family economic status.

### Measures

#### Cognitive Ability

There are cognitive ability test questions for students in CEPS. Test questions were constructed based on the Taiwan Education Panel Survey (TEPS) (59). According to testing principles, the project team formed a special test group and conducted a trial test in two middle schools in Shanghai and Zhengzhou, China. After three rounds of revision, the cognitive ability test questions were finally determined. Test questions measure students’ logical thinking and problem-solving skills rather than asking students to recall specific knowledge taught in the school curriculum. The test scores can be compared with international and national standards. A total of 35 questions were developed to measure 8th students’ cognitive ability, including language ability, spatial ability and logical ability (60). Previous studies have demonstrated that this test was valid (61, 62). The time limitation for the test is 30 mins. The scores were standardized using Item Response Theory (IRT) with three parameters (difficulty index, discriminative powder index, guessing index) (3PL) to ensure comparability of scores across the students (60). The more detailed introduction to this cognitive ability test can be found on the website of the CEPS.^1^

#### Extracurricular Activity Participation

Several items in the CEPS Student Questionnaire were used to measure extracurricular activity participation of students. At first, participants were asked: “From Monday to Friday, what is your daily schedule of extracurricular activities?” and “On weekends, what is your daily schedule of extracurricular activities?” The activities under these two questions included “completing homework assigned by teachers,” “attending extracurricular tutoring (related to schoolwork),” “attending interest classes (unrelated to schoolwork),” “watching TV” and “surfing the Internet and playing games.” The answer options for extracurricular activities from Monday to Friday are: 1 = none, 2 = less than 1 h, 3 = about 1–2 h, 4 = about 2–3 h, 5 = about 3–4 h, and 6 = about 4 h or more. The answer options for weekend extracurricular activities are: 1 = none, 2 = less than 2 h, 3 = about 2–4 h, 4 = about 4–6 h, 5 = about 6–8 h, and 6 = about 8 h or more.

In addition, we used the item of “Your physical exercise time: usually ___days a week, ___ minutes a day” to measure students’ physical exercise time. The answers were calculated as students’ weekly physical exercise time and the data were converted into natural logarithmic form.

#### Demographics Variables

We collected data on the demographic and family characteristics of the respondent. Individual student characteristics included gender (female = 0, male = 1), Hukou type (urban Hukou = 0, rural Hukou = 1) and whether they are only children (no = 0, yes = 1). Family characteristics included family economic status and parents’ highest education level. Among them, family economic status was reported by parents in response the question “What is the current economic situation of your family?” This variable was also reported by students in response to the question “How do you feel about your family’s financial situation right now?” Both items were rated using a five-point frequency response scale: 1 = very difficult, 2 = relatively difficult, 3 = fine, 4 = relatively wealthy, and 5 = very wealthy. We used the parent-report data to determine the family financial situation first. When the parent-report data were missing, we used the student-report data. Finally, the responses were merged into three categories: The first category is “families perceived to have financial difficulty (DIFF),” including “very difficult” and “relatively difficult” responses. The second category is “families perceived to have “fine” financial situation (FINE), including the “fine” response. The final category is “families perceived to wealthy (WEALTHY), including “relatively wealthy” and “very wealthy” families.

The parents’ highest education level was to compare the education levels of students’ fathers and mothers and taken the relatively higher value with reference to the following categories: 1 = no education, 2 = primary school, 3 = middle school, 4 = technical secondary school/technical school, 5 = vocational high school, 6 = high school, 7 = college, 8 = undergraduate degree, 9 = graduate and above.

### Data Analysis Strategy

Considering that the level of students’ cognitive ability may be affected by the schools they attended which would lead to the problem of data aggregation, we adopted multilevel regression model (63) to analyze the data. In this study, students were nested in schools, with students at the first level and schools at the second level, and two-level linear model analysis method was adopted. The model form was as follows:


(1) Level⁢ 1:yij=β0⁢i+eij

(2) Level⁢ 2:β0⁢i=γ00+u0⁢i

where γ_00_ is the fixed effect and *u*_0_*_i_* + e*_ij_* is the random effect. Results based on the intercept model (see Supplementary Table 1) showed that the intragroup correlation coefficient (ICC) was 0.298, which indicated that 30% of the total variation of students’ cognitive ability was due to differences among different schools (63). As such, we used the SPSS multilevel regression model (i.e., mixed linear modeling) in our analyses.

In the multilevel regressions, Model 1 included only the controlled variables, and we added all extracurricular activities in Model 2. Moreover, to investigate the moderating effects of gender and family economic status on the relationships between extracurricular activities and cognitive ability among junior high school students, we added interaction terms for extracurricular activities (Model 3) and gender (Model 4). We conducted all analyses in SPSS 21.0.

## Results

### Descriptive and Correlation Analyses

Table 1 shows the descriptive statistics of our sample. The average score for student cognitive ability estimated with the three-parameter IRT model was 0.29. In all extracurricular activities except physical exercise, students spent most time completing homework assigned by teachers (*M*_weekdays_ = 3.51; *M*_weekends_ = 3.00) and least time on attending interest classes (*M*_weekdays_ = 1.38; *M*_weekends_ = 1.38).

**TABLE 1:** Means and standard deviations of all variables.

Variables	Range	*M* (SD)
**Dependent variable**		
Cognitive ability	−3.14 to 2.06	0.29 (0.83)
**Independent variable (weekdays)**		
Completing homework assigned by teachers	1–6	3.51 (1.15)
Attending extracurricular tutoring (related to schoolwork)	1–6	1.60 (1.23)
Attending interest classes (unrelated to schoolwork)	1–6	1.38 (0.92)
Watching TV	1–6	2.47 (1.41)
Surfing the Internet and playing games	1–6	2.25 (1.44)
**Independent variable (weekends)**		
Completing homework assigned by teachers	1–6	3.00 (1.04)
Attending extracurricular tutoring (related to schoolwork)	1–6	1.69 (1.15)
Attending interest classes (unrelated to schoolwork)	1–6	1.38 (0.82)
Watching TV	1–6	2.76 (1.21)
Surfing the internet and playing games	1–6	2.61 (1.36)
Engaging in physical exercise	0–8.85	4.69 (0.91)
**Control variables**		
Gender	0–1	0.52 (0.50)
Hukou type	0–1	0.52 (0.50)
Family economic status	1–3	1.85 (0.50)
Only child status	0–1	0.55 (0.50)
Parents’ highest educational level	1–9	4.60 (2.03)

Correlation analyses showed that for most extracurricular activities, time spent on the activity was significantly correlated with cognitive ability but with low effect size (see Table 2). Among them, time spent completing homework assigned by teachers on weekdays and weekends, attending extracurricular tutoring, interest classes on weekends and physical exercise were positively correlated with cognitive ability (*r* = 0.024, *p* < 0.05–0.193, *p* < 0.001), while time spent attending interest classes on weekdays, watching TV and playing games online were negatively correlated with cognitive ability (*r* = −0.026, *p* < 0.05 to −0.241, *p* < 0.001).

**TABLE 2:** Correlation between extracurricular activities and cognitive ability.

	Cognitive ability
**Weekdays**	
Completing homework assigned by the teacher	0.193***
Attending extracurricular tutoring (related to schoolwork)	–0.011
Attending interest class (unrelated to schoolwork)	−0.047***
Watching TV	−0.241***
Surfing online and playing games.	−0.177***
**Weekends**	
Completing homework assigned by the teacher	0.193***
Attending extracurricular tutoring (related to schoolwork)	0.152***
Attending interest class (unrelated to schoolwork)	0.024*
Watching TV	−0.100***
Surfing online and playing games.	−0.026**
Physical exercise	0.159***
**Control variable**	
Gender	−0.051***
Hukou type	−0.134***
Family economic status	0.147***
Only child status	−0.181***
Parents’ highest educational level	0.262***

*p < 0.05, **p < 0.01, and ***p < 0.001.

### Relationships Between Extracurricular Activities and Cognitive Ability

Results of the multilevel regression analyses on the relationships between extracurricular activities and cognitive ability are shown in Table 3. Results of Model 1 indicated that the cognitive ability of girls was higher than that of boys (*M*_boys_ = 0.27, *M*_girls_ = 0.36, β = −0.070, *p* < 0.001). In addition, the parents’ highest education level had a small but significant positive relationship with students’ cognitive ability (β = 0.038, *p* < 0.001).

**TABLE 3:** Fixed effect model of extracurricular activities on junior high school students’ cognitive ability.

	Model 1	Model 2a	Model 2b

	β	β (weekdays)	β (weekends)
**Control variable**			
Gender	−0.070***	−0.047**	−0.056***
Hukou type	0.013	0.010	0.006
Family economic status	0.017	0.018	0.017
Only child status	–0.032	0.020	−0.024***
Parents’ highest educational level	0.038***	0.035***	0.034***
**Independent variable**			
Completing homework assigned by the teacher		0.059***	0.063***
Attending extracurricular tutoring (related to schoolwork)		−0.032***	–0.000
Attending interest class (unrelated to schoolwork)		−0.067***	−0.069***
Watching TV		−0.055***	−0.018**
Surfing online and playing games.		−0.046***	−0.015*
Physical exercise		0.070***	0.069***
Sample size	9,830		

*p < 0.05, **p < 0.01, and ***p < 0.001. Model 1 only included a series of control variables, and Model 2a and Model 2b added the variables of participation in extra-curricular activities on weekdays and weekends, respectively.

The predictor variables were added to the Model 2 based on Model 1 (Model 2a was participation of extracurricular activities on weekdays and Model 2b was participation of extracurricular activities on weekends). Results showed that time spent on completing homework assigned by teachers on weekdays and weekends was positively related to students’ cognitive ability (β = 0.059, *p* < 0.001 and β = 0.063, *p* < 0.001, respectively). Time spent attending extracurricular tutoring (related to schoolwork) on weekdays was negatively associated with students’ cognitive ability (β = −0.032, *p* < 0.001), while time spent attending extracurricular tutoring on weekends had no significant relationship with students’ cognitive ability. Additionally, time spent attending interest classes (unrelated to schoolwork) on weekdays and weekends was negatively associated with students’ cognitive ability (β = −0.067, *p* < 0.001; β = −0.069, *p* < 0.001), and time spent taking part in physical exercise on weekdays and weekends had a significant positive relationship with students’ cognitive ability (β = 0.070, *p* < 0.001; β = 0.069, *p* < 0.001). In short, Hypothesis 1a was partially supported.

In terms of recreational activities, time spent watching TV on weekdays and weekends was negatively associated with students’ cognitive ability, β = −0.055, *p* < 0.001; β = −0.018, *p* < 0.001. In addition, time spent surfing the Internet and playing games also was negatively associated with students’ cognitive ability (weekdays: β = −0.046, *p* < 0.001 and weekends: β = −0.015, *p* < 0.05, respectively). Hypothesis 1b was supported.

### Moderating Effect of Gender

Results in Table 4 show significant interaction effect between time spent completing homework and attending extracurricular tutoring on weekdays and gender on students’ cognitive ability (β*_homework_* = 0.043, *p* < 0.05; β*_tutoring_* = 0.043, *p* < 0.01, respectively). The simple effect test in Table 5 shows that time spent completing homework assigned by teachers had stronger positive relationship with cognitive ability among boys than did girls (β_boys_ = 0.079, *p* < 0.001; β_girls_ = 0.046, *p* < 0.001); time spent attending extracurricular tutoring on weekdays had stronger negative relationship with cognitive ability among girls than boys (β_girls_ = −0.055, *p* < 0.001; β_boys_ = −0.01, *p* > 0.05). Therefore, Hypotheses 2a were not supported. Additionally, the interaction effect between time spent on watching TV, surfing online and playing online games and gender on student cognitive ability were not significant, which did not support Hypothesis 2b.

**TABLE 4:** Fixed effect model of extracurricular activities on junior high school students’ cognitive ability (adding interaction variables).

	Model 3a	Model 3b	Model 4a	Model 4b

	β (weekdays)	β (weekend)	β (weekdays)	β (weekend)

Control variable	Controlled	Controlled	Controlled	Controlled
**Independent variable**				
Completing the homework assigned by the teacher	0.038***	0.054***	0.083***	0.099***
Attending extracurricular tutoring (related to schoolwork)	−0.052***	–0.007	−0.035*	–0.014
Attending interest class (unrelated to schoolwork)	−0.055***	−0.058***	−0.058*	−0.127***
Watching TV	−0.054***	–0.016	−0.065***	−0.046**
Surfing online and playing games.	−0.039***	–0.013	−0.044***	0.005
Physical exercise	0.099***	0.100***	0.111***	0.124***
**Extracurricular activities × Gender**				
Completing the homework assigned by the teacher × Gender	0.043*	0.022	–	–
Attending extracurricular tutoring (related to schoolwork) × Gender	0.043**	0.014	–	–
Attending interest class (unrelated to schoolwork) × Gender	–0.029	–0.025	–	–
Watching TV × Gender	–0.005	–0.004	–	–
Surfing the Internet and playing games × Gender	–0.009	–0.000	–	–
Physical exercise × Gender	−0.044*	−0.045*	–	–
**Extracurricular activities × Family economic status**				
Completing the homework assigned by the teacher × (FINE vs DIFF)			–0.027	–0.038
Attending extracurricular tutoring (related to schoolwork) × (FINE vs DIFF)			0.006	0.021
Attending interest class (unrelated to schoolwork) × (FINE vs DIFF)			–0.007	0.074*
Watching TV × (FINE vs DIFF)			0.023**	0.035*
Surfing the Internet and playing games × (FINE vs DIFF)			0.006	–0.025
Physical exercise × (FINE vs DIFF)			−0.053**	−0.068**
Completing homework assigned by the teacher × (WEALTH vs DIFF)			–0.030	–0.019
Attending extracurricular tutoring (related to schoolwork) × WEALTH vs DIFF)			0.002	–0.006
Attending interest class (unrelated to schoolwork) × (WEALTH vs DIFF)			−0.076*	0.021
Watching TV × (WEALTH vs DIFF)			0.043	0.016
Surfing the Internet and playing games × (WEALTH vs DIFF)			–0.031	–0.012
Physical exercise × (WEALTH vs DIFF)			–0.047	–0.069
Sample size	9,830		9,830

*p < 0.05, **p < 0.01, and ***p < 0.001. Two virtual variables were generated for family economic status: FINE vs DIFF and WEALTH vs DIFF. The reference group is the DIFF group.

**TABLE 5:** Gender differences in the fixed effects of extracurricular activities on cognitive ability.

	Weekdays		Weekends	
		
	β (boys)	β (girls)	β (boys)	β (girls)
**Control variable**				
Hukou type	0.000	0.010	0.006	0.015
Family economic status	0.030	0.026	0.029	0.024
Only child status	–0.039	–0.022	−0.059*	–0.007
Parents’ highest educational level	0.041***	0.035***	0.037***	0.036***
**Independent variable**				
Completing homework assigned by the teacher	0.079***	0.046***	0.070***	0.064***
Attending extracurricular tutoring (related to schoolwork)	–0.010	−0.055***	0.018	0.001
Attending interest class (unrelated to schoolwork)	−0.082***	−0.045***	−0.084***	−0.036*
Watching TV	−0.056***	−0.061***	−0.019*	–0.017
Surfing online and playing games.	−0.048***	−0.035***	–0.016	–0.011
Physical exercise	0.055***	0.106***	0.055***	0.109***

*p < 0.05, ***p < 0.001.

Moreover, the interaction effect of time spent on physical exercise and gender on students’ cognitive ability was also significant (β_weekdays_ = −0.044, *p* < 0.05; β_weekends_ = −0.045, *p* < 0.05, respectively; see Table 4). The simple effect test in Table 5 shows that time spent on physical exercise showed stronger positive relationship with girls’ cognitive ability than did boys (weekdays: β_boys_ = 0.055, *p* < 0.001, β_girls_ = 0.106, *p* < 0.001; weekend: β_boys_ = 0.055, *p* < 0.001, β_girls_ = 0.109, *p* < 0.001) which was opposite to the prediction in Hypothesis 2c.

### Moderating Effect of Family Economic Status

The interaction term in Table 4 shows that the effect of time spent attending interest class on weekdays and weekends on students’ cognitive ability was moderated by family economic status (weekdays: β_WEALTH vs DIFF_ = −0.076, *p* < 0.05; weekends: β_FINE vs DIFF_ = 0.074, *p* < 0.05). Table 6 further shows that time spent attending interest classes on weekdays had a stronger negative relationship with the cognitive ability among students from the “WEALTH” category than did students from the “DIFF” category (β_WEALTH_ = −0.129, *p* < 0.001; β_DIFF_ = −0.054, *p* < 0.05), and time spent interest class on weekends had a stronger negative relationship with the cognitive ability among students from students in the “DIFF” category than did students from the “FINE” category (β_DIFF_ = −0.133, *p* < 0.001; β_FINE_ = −0.056, *p* < 0.001). Hypothesis 3a was not supported.

**TABLE 6:** Fixed effects of extracurricular activities on cognitive ability in different family economic status groups.

	Weekdays			Weekends		
		
	Difficult	Moderate	Wealthy	Difficult	Moderate	Wealthy
**Control variable**						
Gender	–0.018	−0.062**	0.055	–0.029	−0.068**	0.025
Hukou type	–0.053	0.027	–0.070	–0.047	0.020	–0.081
Only child status	–0.037	–0.022	–0.098	–0.033	–0.028	–0.066
Parents’ highest educational level	0.050***	0.037***	0.051**	0.054***	0.034***	0.056**
**Independent variable**						
Completing homework assigned by teachers	0.088***	0.057***	0.077**	0.107***	0.058***	0.090**
Attending extracurricular tutoring (related to schoolwork)	–0.038	−0.027**	–0.033	–0.039	0.020*	–0.010
Attending interest class (unrelated to schoolwork)	−0.054*	−0.064***	−0.129***	−0.133***	−0.056***	−0.099**
Watching TV	−0.091***	−0.047***	–0.029	−0.048**	–0.012	–0.047
Surfing online and playing games.	–0.022	−0.052***	−0.082**	–0.006	−0.021**	0.009
Physical exercise	0.112***	0.066***	0.074*	0.129***	0.061***	0.065

*p < 0.05, **p < 0.01, and ***p < 0.001.

Moreover, we found that significant interaction effect between time spent watching TV on weekdays and weekends and family economic status on students’ cognitive ability (weekdays: β_FINE vs DIFF_ = 0.023, *p* < 0.01; weekends: β_FINE vs DIFF_ = 0.035, *p* < 0.025). Table 6 further shows that the negative relationship of time spent watching TV on weekdays with the cognitive ability among students from the “DIFF” category was stronger than that among students from the “FINE” category (weekdays: β_DIFF_ = −0.091, *p* < 0.001; β_FINE_ = −0.047, *p* < 0.001). Besides, while time spent watching TV on weekends had a negative relationship with the cognitive ability among students from the “DIFF” category, the relationship was not significant for students from the “FINE” group (weekends: β_DIFF_ = −0.048, *p* < 0.01; β_FINE_ = −0.012, *p* > 0.05). Hence, Hypothesis 3b was partly supported.

In addition, significant interaction between time spent on physical exercise and family economic status on students’ cognitive ability was also found (weekdays: β_FINE vs DIFF_ = −0.053, *p* < 0.01; weekends: β_FINE vs DIFF_ = −0.068, *p* < 0.01). Further analysis shows that time spent on physical exercise had a stronger positive relationship with cognitive ability among students from the “DIFF” category than did students from the “FINE” category (weekdays: β_DIFF_ = 0.112, β_FINE_ = 0.066; weekends: β_DIFF_ = 0.129, β_FINE_ = 0.061). Hypothesis 3c was supported. In summary, we present the outcomes of the different hypotheses in Table 7.

**TABLE 7:** Summary of the support for the different hypotheses.

Hypotheses	Support or not
**Hypothesis 1a:** time spent on homework, extracurricular tutoring, interest classes and physical exercise would be positively associated with cognitive ability	Partially supported
**Hypothesis 1b:** time spent on watching TV, surfing the Internet and playing games would be negatively associated with cognitive ability	Supported
**Hypothesis 2a:** time spent on homework, extracurricular tutoring and interest classes would be more strongly related to the cognitive ability among girls than boys in a positive manner	No
**Hypothesis 2b:** time spent on watching TV, surfing online and playing online games would be more strongly associated with cognitive ability in boys than girls in a negative manner	No
**Hypothesis 2c:** positive relationship between the duration of extracurricular physical exercise and cognitive ability would be stronger in boys than in girls	No, contrary to hypothesis
**Hypothesis 3a:** relative to adolescents with high family economic status, time spent on homework, extracurricular tutoring and interest classes would have stronger relationship with cognitive ability among students with low family economic status	No
**Hypothesis 3b:** time spent on watching TV and playing games online would have a stronger negative relationship with cognitive ability among students with low family economic status than did students with high family economic status	Partially supported
**Hypothesis 3c:** time spent on physical exercise would have a stronger positive relationship with cognitive ability among students with low family economic status than did students with high family economic status	Supported

## Discussion

Our findings indicated that time spent completing homework assigned by teachers and physical exercise was positively related to cognitive ability among junior high school students, while time spent attending extracurricular tutoring on weekdays, attending interest class, watching TV and surfing online and playing games had significant negative relationships with students’ cognitive ability. Furthermore, there is some support for the moderating effect of gender and family economic status. These are pioneer findings in different Chinese contexts.

The present findings showed that time spent completing homework assigned by teachers was positively related to students’ cognitive ability, which is consistent with the previous findings (19–21). However, time spent attending extracurricular tutoring on weekdays was negatively associated with students’ cognitive ability, and time spent attending extracurricular tutoring on weekends was not related to students’ cognitive ability. These findings are inconsistent with previous research findings (27–29). This puzzle may possibly be explained by the unique Chinese cultural characteristics. Influenced by the strong cultural emphasis on academic excellence, Chinese parents typically attach great importance to children’s education success and regard it as “glory” of the whole family (64). Academic achievement is an obvious indicator of success of education. In order to improve their children’s academic achievement, Chinese parents try their best to get their children to attend various extracurricular tutoring classes. According to many studies, the number of Chinese primary and secondary school students participating in extra-curricular tutoring exceeded 100 million in 2016 (65). However, in reality, an average student needs to spend more than 8 h in school a day from Monday to Friday, and the school teaching has made his/her acceptance and energy reach a “saturated” state. Therefore, arranging extra tutoring for students on weekdays may have an adverse effect because it may create cognitive and emotional exhaustion for the students. In addition, compared with extracurricular tutoring, improvement of students’ cognitive ability may be influenced by other factors, such as time investment in family education (66). Furthermore, we found that attending interest classes was negatively related to students’ cognitive ability, which is also contrary to our hypothesis. In future studies, we have to investigate whether students choose to join the interest classes in a voluntary manner. As it is common that Chinese parents “order” the children to join interest classes, joining such classes may lead to resentment of the students.

We found that excessive TV watching, surfing online and playing games were negatively associated with students’ cognitive ability, which is consistent with previous research findings (34, 38, 53, 67). The development of individual thinking is related to the formation of abstract thinking and comprehensive cognitive processing of left and right brains. The intuitive images of television presentation are not conducive to the formation and development of children’s abstract thinking, nor to the interaction between children’s left and right brains. Therefore, watching TV excessively will weaken the development of students’ thinking ability, thus leading to the decline of cognitive ability (68). Besides, studies have shown that addiction to the Internet makes individuals less efficient with regard to information processing and response inhibition, and their executive control ability and capacity for attention may also be impaired (35), which is also the reason for the decline of cognitive ability. Furthermore, excessive TV and Internet consumption may also imply a lack of parental supervision which indirectly lead to poorer developmental outcomes, such as cognitive ability.

Finally, we found that time spent on physical exercise was positively related to students’ cognitive ability, which is consistent with previous findings (39–42). Previous studies have shown that regular physical exercises can improve children’s and adolescents’ brain structure and cognitive functions (69). As Chinese education emphasizes much on academic excellence and deemphasizes “play,” the present findings constitute “food for thought” for parents. In particular, faced by COVID-19 where there are restriction of social activities (70), how physical exercise may promote the holistic development of adolescents is an important issue to be considered.

### The Moderating Effects of Adolescents’ Gender

We found moderating effects of gender on the association between participation in some extracurricular activities and cognitive ability, although the findings are not in line with our original hypotheses based on past studies. First, time spent completing homework assigned by teachers had stronger positive relationship with cognitive ability in boys than girls. This finding is consistent with Kalenkoski and Pabilonia (71) finding that time spent on homework had no effect on the long-term academic achievement of high school girls but it positively contributed to the long-term academic achievement of boys, such as substantial increase in the probability of college attendance for boys. The present finding can possibly be explained by the notion of “marginal return in cognitive ability” (40). Previous studies showed that female students reported spending more effort or being more persistent on homework (72, 73) and using more planning or monitoring strategy (74) than did male students. In addition, girls’ academic performance is generally better than boys’ (47, 75). As a result, girls’ improvement of academic performance or cognitive ability through regular homework may be constrained by “reduced marginal returns,” while the lower baseline may enables boys to get higher improvement return of academic performance or cognitive ability from homework.

Additionally, we found that time spent attending extracurricular tutoring on weekdays had stronger negative relationship with the cognitive ability among girls than boys. One possible explanation is that as girls spend more time and effort in school learning than did boys (47), girls may suffer from more negative impacts of extracurricular tutoring on cognitive ability compared with boys if there is “additional” academic demands in the form of extracurricular tutoring during weekdays. Regarding the stronger positive relationship between physical exercise and cognitive development in girls, we can also use the “marginal return” explanation. That is, as the intensity and frequency of physical exercise for girls are lower than that for boys (76, 77), girls may get more obvious marginal returns for improving their cognitive ability by taking part in physical exercise because they may start with a lower baseline.

However, we found that the moderating effects of gender were not significant on the relationships of watching TV and playing games online with cognitive ability, which was inconsistent with our hypothesis. Although most previous studies indicated that boys spent more time on the Internet, had higher risk of Internet addiction, and might be more vulnerable to negative effects (50–53), a few studies indicated that Internet use and playing video games made boys outperformed girls in visual-spatial skills, while girls outperformed boys in standardized tests of reading skills (78). Based on 479 9th grade students in Turkey, Dindar (79) found that although boys spent more time playing video games, this did not lead to gender differences in problem solving competence. These findings suggest that the role of gender on the relationship between screen time and cognitive ability may not be simple. For example, some studies showed that gender differences in the effects of screen time use may be due to depression (80, 81).

In short, as the findings on the moderating effect of gender are not conclusive, there is a need to conduct more studies in future. Nevertheless, the present findings form the bases for developing testable hypotheses for future studies.

### The Moderating Effects of Family Economic Status

We also found that family economic status moderated the association between participation in extracurricular activities and cognitive ability. Specifically, time spent watching TV on weekdays and weekends showed a stronger negative relationship with cognitive ability among students with reported family financial difficulty as compared with students experiencing “fine” family financial status. This observation is consistent with previous study (82). From the perspective of self-worth orientation (83), the more self-worth support resources a person had, such as high social status, high income, outstanding achievements, etc., the less likely he/she would be attracted by self-worth resources that were not recognized by society, such as turning to non-performing groups and indulging in the virtual world. Previous studies have shown that higher family socioeconomic status of adolescents was positively related to self-worth (84) and the lower socio-economic status children reported more screen time (85). Generally, a higher level of socio-economic well-being of the family and the opportunity to provide children with additional activities are associated with shorter screen time (86). On the other hand, students with lower family economic status are weaker than those with higher family economic status in self-worth support resources recognized by society and the opportunity to participate additional activities. As a result, low-SES children were more likely to spend more time on screen and get satisfaction in the virtual world (i.e., compensation). Moreover, as children with low family socioeconomic status are generally associated with low parental involvement (87), low-SES parents are less likely to buy learning materials for their children, take their children to cultural events, and control their children’s TV time (88). Therefore, students from low-SES family were more likely to become addicted to TV or Internet, which would have negative impact on their cognitive ability.

In terms of physical exercise, compared with students from the “FINE” and “WEALTHY” groups, time spent on physical exercise was positively related to cognitive ability among students from the “DIFF” group in a stronger manner. Research has shown that low-SES students lack the materials and experiences to obtain cognitive stimulation (88) which limits their participation in extracurricular activities compared with students with high family economic status. As such, taking part in physical exercise may be an effective way to improve their cognitive ability. Through physical activities (such as ball games which require problem solving skills), there are opportunities nurturing the cognitive abilities of students experiencing financial difficulty. Future studies should be conducted to examine this conjecture further.

### Strengths and Limitations

This study has important contributions to the literature by examining the relationships between unstructured extracurricular activities and early adolescents’ cognitive ability and exploring the moderating effects of adolescents’ gender and family economic status. First, the current study replicated previous findings showing the positive relationships of homework and physical exercise and negative relationships of excessively watching TV and surfing online to adolescent cognitive ability. Second, the present study suggests the negative relationship between extra-curricular tutoring and interest classes and cognitive ability among Chinese adolescents, which challenge the previous research results. Third, these findings contribute to our understanding of gender and family economic status as the moderators. Finally, this study used a large sample and multi-level multiple regression analyses which could improve the internal validity of the findings.

However, the present study has some limitations. First, this study used cross-sectional survey data, which may not be as powerful as longitudinal studies in terms of explanatory power. For example, we cannot test the cause-effect relationships involved because both variables take place at the same time. In future, we can consider using longitudinal data to analyze the causal relationship between participation in extracurricular activities and students’ cognitive ability. Second, data in this study were collected using only students’ self-report questionnaires, which may influence the results due to response bias. Future research should gather information from both parents and adolescents. Third, in the present study, extracurricular activities were measured by using questionnaires from the China Education Panel Survey, which could not be compared with other relevant studies. Fourth, other important measures such as “sleep” was not included in this study, which may be negatively affected by extracurricular activities and lead to negative cognitive outcome. In the future, students’ sleep should be included in the research. Fifth, it would also be interesting in the future to compare these results using academic performance such as grades. It is theoretically possible that tutoring and extra classes may lead to better grades but not better critical thinking or creativity. Arts and creative classes, on the other hand, may enhance creative thinking as part of a child’s cognitive skills. Finally, as the effect size of our results was low, the statistically significant finding may simply be due to the large sample size. There are distinctions between statistical significance and practical significance (89), where statistical significance could be influenced by sample size (90). Thus, we should be cautious when interpreting these results.

### Practical Implications

The present findings provide information for parents when they plan extra-curricular activities for their children. First, they should rationally treat extracurricular tutoring and interest classes. We have found that time spent attending extracurricular tutoring and interest classes had significant negative correlations with students’ cognitive ability. So when arranging such extracurricular activities for their children, they could consider children’s acceptance and make reasonable arrangements to avoid adverse effects of such activities on children’s cognitive ability. In particular, there are studies showing that excessive extra-curricular activities were related to adolescent depression (91, 92). Second, parents should reasonably control their children’s time for watching TV, surfing online and other entertainment activities, and help them cultivate self-control ability. Especially, parents with lower family economic status should pay more attention to the extracurricular entertainment activities of adolescents. Third, adolescents should be aware of the benefits of physical exercise and develop the habit of physical exercise. As the findings identified some gender differences, schools and parents can specially organize sports suitable for girls, encourage them to actively participate in sports activities.

In terms of public policies on youth development, although the Chinese Government has issued policies to rigorously regulate the extracurricular training market (93), it also needs to strengthen the supervision of the implementation and effect of these policies to ensure that extracurricular training institutions can bring positive influence to students’ development. Secondly, the government should also increase investment in the construction of public facilities such as places for extracurricular activities of adolescents, such as youth palaces and sports venues, so as to create favorable conditions for children and adolescents to participate in extracurricular activities. Third, as life skills and positive youth development programs are important to the holistic development of adolescents (94–96), promotion of such programs can help to promote the holistic development of Chinese adolescents.

## Conclusion

Consistent with the literature, this study showed that time spent completing homework and physical exercise were positively related to early adolescents’ cognitive ability, while time spent attending extracurricular tutoring, interest classes, watching TV, and surfing online and playing games were negatively related to students’ cognitive ability. Second, moderating effect of gender and family economic status on the relationship between participation in extracurricular activities and early adolescents’ cognitive ability were found. Specifically, time spent on completing homework assigned by teachers had stronger positive correlation with boys’ cognitive ability, while time spent attending extracurricular tutoring on weekdays had stronger negative relationship with cognitive ability among girls than boys, and time spent on physical exercise showed stronger positive correlation with cognitive ability among girls than boys. Besides, the negative correlation between time spent attending interest classes on weekdays and cognitive abilities was stronger in students from reported wealthy families relative to students experiencing financial difficulty. Finally, compared to students from economically advantaged families, time spent watching TV had stronger negative relationships with the cognitive ability among students from families with perceived financial difficulty. At the same time, stronger positive relationship between physical exercise and cognitive ability in students experiencing family financial difficulty relative to students experiencing better family financial condition.

## Data Availability Statement

The raw data supporting the conclusions of this article will be made available by the authors, without undue reservation.

## Ethics Statement

The studies involving human participants were reviewed and approved by Research Ethics Committee of Renmin University of China. Written informed consent to participate in this study was provided by the participants’ legal guardian/next of kin.

## Author Contributions

YP and DZ: conceptualization, formal analysis, and writing—original draft preparation. YP and DS: methodology and writing—review and editing. All authors have read and agreed to the publication of this manuscript.

## Conflict of Interest

The authors declare that the research was conducted in the absence of any commercial or financial relationships that could be construed as a potential conflict of interest.

## Publisher’s Note

All claims expressed in this article are solely those of the authors and do not necessarily represent those of their affiliated organizations, or those of the publisher, the editors and the reviewers. Any product that may be evaluated in this article, or claim that may be made by its manufacturer, is not guaranteed or endorsed by the publisher.

## Footnotes

1
http://ceps.ruc.edu.cn/
