# Supplementary material for: Participation in After-School Extracurricular Activities and Cognitive Ability Among Early Adolescents in China: Moderating Effects of Gender and Family Economic Status
Source: Front Pediatr. 2022 Mar 17;10:839473. doi: 10.3389/fped.2022.839473 (PMC8968855; doi:10.3389/fped.2022.839473)
Supplement: Supplementary file 1 [file Table_1.docx]

Table S1- Covariance parameter estimation

| Parameter | Estimate | *SE* | *P* |
| --- | --- | --- | --- |
| residual | .499 | .007 | <.001 |
| intercept [individual =school] variance | .212 | .029 | <.001 |
